# One strategy for arthroscopic suture fixation of tibial intercondylar eminence fractures using the Meniscal Viper Repair System

**DOI:** 10.1186/1758-2555-3-17

**Published:** 2011-08-10

**Authors:** Satoshi Ochiai, Tetsuo Hagino, Yoshiyuki Watanabe, Shinya Senga, Hirotaka Haro

**Affiliations:** 1 The Sports Medicine and Knee Center, National Hospital Organization, Kofu National Hospital, 11-35 Tenjin-cho, Kofu, Yamanashi 400-8533, Japan; 2 Department of Orthopaedic Surgery, Faculty of Medicine, University of Yamanashi, Yamanashi, Japan

## Abstract

**Background:**

Principles for the treatment of tibial intercondylar eminence fracture are early reduction and stable fixation. Numerous ways to treatment of this fracture have been invented. We designed a simple, low-invasive, and arthroscopic surgical strategy for tibial intercondylar eminence fracture utilizing the Meniscal Viper Repair System used for arthroscopic meniscal suture.

**Methods:**

We studied 5 patients, who underwent arthroscopic suture fixation that we modified. The present technique utilized the Meniscal Viper Repair System for arthroscopic suture of the meniscus. With one handling, a high-strength ultra-high molecular weight polyethylene(UHMWPE) suture can be passed through the anterior cruciate ligament (ACL) and the loops for suture retrieval placed at both sides of ACL. Surgical results were evaluated by the presence or absence of bone union on plain radiographs, postoperative range of motion of the knee joint, the side-to-side differences measured by Telos SE, and Lysholm scores.

**Results:**

The reduced position achieved after surgery was maintained and good function was obtained in all cases. The mean distance of tibia anterior displacement and assessment by Lysholm score showed good surgical results.

**Conclusion:**

This method simplified the conventional arthroscopic suture fixation and increased its precision, and was applicable to Type II fractures that could be reduced, as well as surgically indicated Types III and IV. The present series suggested that our surgical approach was a useful surgical intervention for tibial intercondylar eminence fracture.

## Background

Tibial intercondylar eminence fracture is an intra-articular avulsion fracture of the anterior cruciate ligament (ACL) insertion, which was first reported by Poncet in 1875 [1]. Because of its unique morphology of injury, the fracture is also considered to be a subtype of ACL injury [2]. If the displacement of the fractured fragment is left uncorrected, complications such as limitation of knee extension, nonunion, and malunion may occur [3,4]. Besides, laxity of ACL may lead to ACL failure symptoms such as anterior instability of the knee [5]. Therefore, in treating this fracture, early reduction and stable fixation are important. We designed a simple, low-invasive, surgical method for tibial intercondylar eminence fracture by (1) improving the pull-out fixation technique, which was one of the representative methods, (2) using the Meniscal Viper Repair System (Arthrex, Naples, FL) for arthroscopic suture of the meniscus, and (3) using ultra-high molecular weight polyethylene (UHMWPE) suture to obtain more stable fixation [6]. We report the methods and surgical results.

## Methods

We studied 5 patients (5 fractures) diagnosed with tibial intercondylar eminence fracture, who underwent arthroscopic suture fixation using the Meniscal Viper Repair System at the Sports Medicine and Knee Center, Kofu National Hospital between February 2007 and October 2009. There were 4 males and 1 female. The ages at surgery ranged from 7 to 55 years (mean 28.8 years). The fracture type according to Meyers et al. and Zaricznyj was type II in 1 patient, type III in 3 patients, and type IV in 1 patient [7,8]. Surgical results were evaluated by the presence or absence of bone union on plain radiographs, postoperative range of motion of the knee joint, the side-to-side differences measured by Telos SE (Telos Technology, Inc., Hamburg, Germany), and pre- and post-operative Lysholm scores.

### Surgical Technique

**Figure 1:**
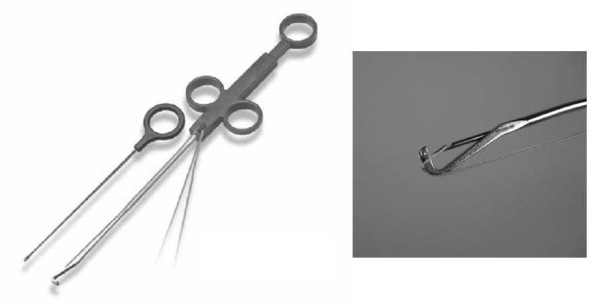
**The Meniscal Viper Repair system**. This device is developed for all-inside meniscal suture. It has a low profile shaft. The distance of the tissue penetration of a needle is long.

The Meniscal Viper Repair System used in this study is a device that has enabled all-inside arthroscopic meniscus suturing. The needle passing through the meniscal tear captures the suture set at the tip of the hook-shaped device (Figure 1).

Surgery was performed under lumbar or general anesthesia. Patients were positioned supine, and a supporting plate was placed at the foot sole and the lateral thigh of affected lower limb. The limb was fixed with the knee flexed at 90° and the ankle joint at neutral position.

Two portals of anteromedial and anterolateral were used for arthroscope insertion. A 30-degree oblique arthroscope was applied to observe the interior of the knee joint. For the cases that had concurrent injuries of intra-articular structures such as the meniscus and articular cartilage, the necessary treatments were conducted.

**Figure 2:**
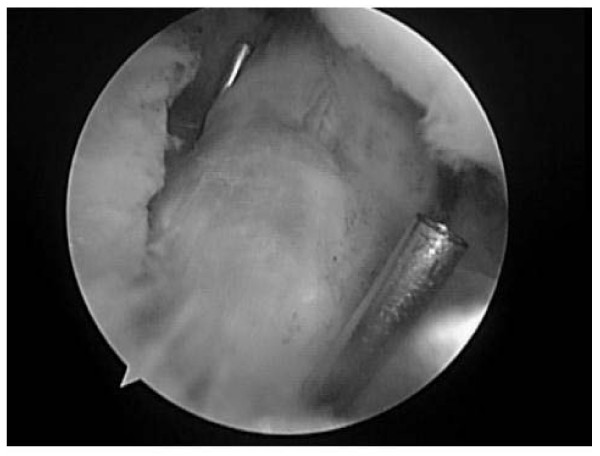
**Insert the suture retriever toward the medial and lateral sides of ACL**. A K-wire was inserted from the antero-medical side of the proximal tibia toward the two sides of the fracture site, to create a drill hole. Two suture retrievers were passed through this drill hole.

At the site of tibial intercondylar eminence fracture, after sufficiently debriding the avulsed bone fragment, the fracture was assessed and confirmed to be capable of reduction. Moreover, it was confirmed that the damage of the main body of ACL is not severe. A transverse skin incision of 1.5 cm was made on the antero-medial side of proximal tibia. Using an ACUFEX director drill guide (Smith & Nephew, Andover, MA), which was a tibial drill hole guide employed in ACL reconstruction, a k-wire in 2.0 mm diameter was inserted toward the medial and lateral sides of fracture site to create a drill hole. After removing the K-wire, a suture retriever (Smith & Nephew, Andover, MA) was inserted into the drill hole (Figure 2), and the loop for suture retrieval was pulled out from the medial and lateral sides of ACL.

**Figure 3:**
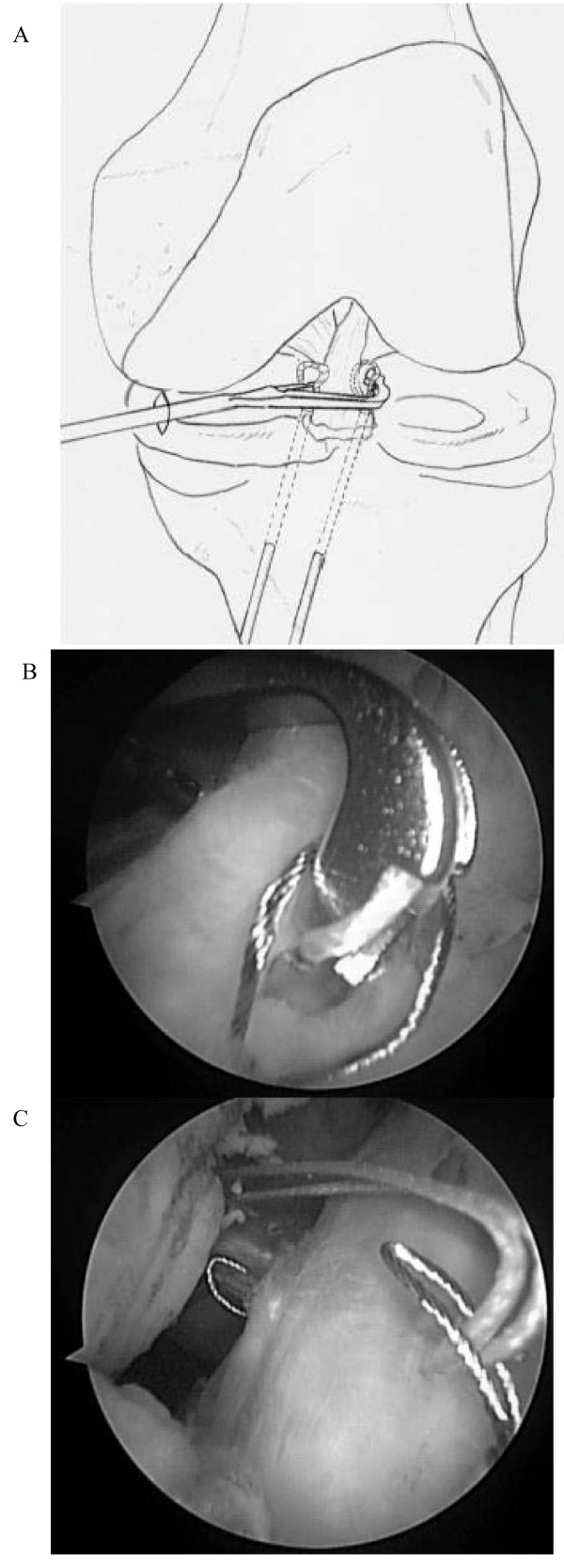
**Suture technique using the Meniscal Viper Repair System**. Using the Meniscal Viper, a UHMWPE suture was passed through in the following order: the loop of suture retriever on the paramedical side of ACL, the distal body of ACL, and the ring on the paralateral side of ACL.

**Figure 4:**
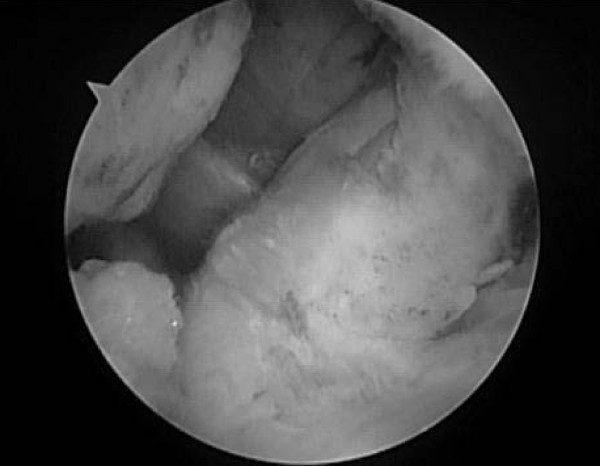
**Suture fixation**. The suture retriever was withdrawn from the drill hole, and the suture was drawn to the antero-medial surface of the proximal tibia. Traction was applied to the suture to reduce and fix the fracture site.

Subsequently, the Meniscal Viper was inserted into the joint via the anteromedial portal. With handling the tip and needle of Meniscal Viper, a UHMWPE suture [2-0 FiberWire (Arthrex) or No. 2 ULTRABRAID (Smith & Nephew)] was passed through in the following order: the suture retriever loop on the paramedial side of ACL, the distal body of ACL, and the ring on the paralateral side of ACL (Figure 3). After the suture was removed from the Meniscal Viper outside the anteromedial portal, the suture retriever was withdrawn from the drill hole, accordingly drawing the suture to the antero-medial side of the proximal tibia. By repeating these procedures, the suture was passed through ACL several times, and drawn to the antero-medial side of proximal tibia. While monitoring the state of reduction of the fracture under an arthroscopic or fluoroscopic view, traction was applied to the sutures, and the sutures were tied tightly on the antero-medial surface of the proximal tibia (Figure 4).

After surgery, the knee was fixed with a cast for 2 weeks. Thereafter, a knee orthosis was worn and range of motion training was started. Partial and full weight-bearing walking was permitted 5 and 8 weeks after surgery, respectively.

## Results

At the last follow-up (8 to 24 months after surgery, mean 16 months), the reduced position achieved after surgery was maintained and good bone union was obtained in all cases. Range of motion of the knee recovered to the normal range in all patients except one who showed 8-degree limitation of knee extension. In that patient, surgery was conducted after a delay of 15 days following injury. The mean side-to-side difference was 3.6 mm. Assessment by Lysholm score showed good surgical results, with improvement from a mean preoperative score of 4 to a mean postoperative score of 90.7 (Table 1).

**Table 1:** Patient Data

Age at surgery (year)	7 - 55 (mean 28.8)	
Sex	Male: 4, Female: 1	
Follow-up period (month)	8 - 22 (mean 16)	
Meyers & Zaricznyj classification	Type I: 0,	Type II: 1
	Type III: 3,	Type IV: 1
Treatment for concurrent injury	Repair of medial collateral ligament: 1	
	Repair of lateral meniscus: 1	
〈SURGICAL RESULT〉		
Range of motion/the side-to-side difference (°)	ext: 0 - 8 (mean 2.0), flx: 0 - 4 (mean 0.8)	
Telos SE/the side-to-side difference (mm)	2.4 - 3.9 (mean 3.6)	
Lysholm score (point)	90 - 100 (mean 90.7)	
Number of sutures	2 - 6 (mean 4)	
Operative time (min)	64 - 88 (mean 71)	

## Discussion

Meyers' classification of fracture types modified by Zaricznyj is widely used as a guide to decide treatment strategies for tibial intercondylar eminence fractures. Conservative treatments are mostly selected for Types I (minimal or non-displaced) and II (partially displaced or hinged), while surgical interventions are indicated forTypes III (completely displaced) and IV (comminuted), and the fractures of Type II that cannot be manually reduced.

In 1982, McLennan reported the usefulness of endoscopic reduction with an arthroscope, and emphasized its advantages including less invasiveness than open surgery, and rapid recovery of knee functions [9]. Later, van Loon and Marti use a drill guide for knee ligament reconstruction in arthroscopic fixation for tibial intercondylar eminence fractures [10]. Moreover, various arthroscopic fixation techniques such as the pull-out method, and fixations using screw, K-wire and staple have been reported [10-13]. All these techniques aimed at more accurate approach to the fracture site and reducing surgical invasiveness.

Among all the available methods, the arthroscopic pull-out method is the most widely applicable technique, because it can be performed regardless of the bone fragment sizes or comminution degrees, and does not require removal of the internal fixation material [11,14,15]. On the other hand, its surgical procedures are complicated and demand skills in arthroscopic techniques. Furthermore, the strength of fixation using sutures remains a concern. In recent years, various attempts have been made to solve the above demerits, including the usage of intra-articular suture devices and UHMWPE sutures [16-19].

We modified the conventional pull-out method with using the Meniscal Viper Repair System that was a device for all-inside meniscal suture, to develop a useful suture fixation technique. Our method had the following four advantages: 1) Technical intra-articular handlings such as passing and retrieving sutures using forceps or suture cannula were not required; 2) A needle could be passed through the intended site accurately without penetrating other tissues; 3) The procedures could be done by using the standard two-portal approach, with no need for additional portal or switching scopes; and 4) Two sutures could be pierced with a single procedure. This method simplified the conventional pull-out method and increased its precision. The operation time for this method was mean 71 min (range 64-88 min). It was shorter than its time for the conventional pull-out method (mean 94 min), and it was not inferior to its time for the screw fixation method (mean 64 min) at this center [10,11]. This method was applicable to Type II fractures that could be reduced, as well as surgically indicated Types III and IV. However, it was important to bear in mind that the surgery should be changed to other procedures such as ACL reconstruction in the cases of concurrent injury to the ACL body. Although further studies with the larger number of cases were required to confirm the clinical outcomes and complications, the present series suggested that our surgical approach was a useful surgical intervention for tibial intercondylar eminence fracture.

## Competing interests

The authors declare that they have no competing interests.

## Authors' contributions

SO conceived of this surgical method, and drafted the manuscript. HH and TH participated in its design and coordination, and proofread. YW and SS helped to draft the manuscript. All authors read and approved the final manuscript.

## References

[B1] Poncet A Bull. et mem Soc de Chir de Paris I 883 1875

[B2] Tohyama H Kutsumi K Yasuda K Avulsion fracture at the femoral attachment of the anterior cruciate ligament after intercondylar eminence fracture of the tibia Am J Sports Med 2002 30 2 279 82 11912101 10.1177/03635465020300022201

[B3] Berg EE Comminuted tibial eminence anterior cruciate ligament avulsion fractures: failure of arthroscopic treatment Arthroscopy 1993 9 4 446 50 10.1016/S0749-8063(05)80320-2 8216577

[B4] Luger EJ Arbel R Eichenblat MS Menachem A Dekel S Femoral notchplasty in the treatment of malunited intercondylar eminence fractures of the tibia Arthroscopy 1994 10 5 550 1 10.1016/S0749-8063(05)80012-X 7999165

[B5] Sullivan DJ Dines DM Hershon SJ Rose HA Natural history of a type III fracture of the intercondylar eminence of the tibia in an adult. A case report Am J Sports Med 1989 17 1 132 3 10.1177/036354658901700124 2929830

[B6] Arthrex-Orthopaedic Products & Medical Education http://www.arthrex.com accessed 10/4/07

[B7] Meyers MH McKeever FM Fracture of the intercondylar eminence of the tibia J Bone Joint Surg Am 1959 41-A 2 209 22 13630956

[B8] Zaricznyj B Avulsion fracture of the tibial eminence: treatment by open reduction and pinning J Bone Joint Surg Am 1977 59 8 1111 4 591548

[B9] McLennan JG The role of arthroscopic surgery in the treatment of fractures of the intercondylar eminence of the tibia J Bone Joint Surg Br 1982 64 4 477 80 6896515 10.1302/0301-620X.64B4.6896515

[B10] van Loon T Marti RK A fracture of the intercondylar eminence of the tibia treated by arthroscopic fixation Arthroscopy 1991 7 4 385 8 10.1016/0749-8063(91)90009-M 1755888

[B11] Carro LP Suzrez CG Cimiano FG Fractures de la espina tibial en ninos. Fijacion por via attroscopica Rev Ortop Trauma 1992 36 200 3

[B12] Bonin N Jeunet L Obert L Dejour D Adult tibial eminence fracture fixation: arthroscopic procedure using K-wire folded fixation Knee Surg Sports Traumatol Arthrosc 2007 15 7 857 62 10.1007/s00167-006-0284-6 17235617

[B13] Kobayashi S Terayama K Arthroscopic reduction and fixation of a completely displaced fracture of the intercondylar eminence of the tibia Arthroscopy 1994 10 2 231 5 10.1016/S0749-8063(05)80100-8 8003155

[B14] Matthews DE Geissler WB Arthroscopic suture fixation of displaced tibial eminence fractures Arthroscopy 1994 10 4 418 23 10.1016/S0749-8063(05)80193-8 7945638

[B15] Kogan MG Marks P Amendola A Technique for arthroscopic suture fixation of displaced tibial intercondylar eminence fractures Arthroscopy 1997 13 3 301 6 10.1016/S0749-8063(97)90025-6 9195025

[B16] Yip DK Wong JW Chien EP Chan CF Modified arthroscopic suture fixation of displaced tibial eminence fractures using a suture loop transporter Arthroscopy 2001 17 1 101 6 10.1053/jars.2001.7805 11154377

[B17] Hsu SY An easy and effective method for reattaching an anterior cruciate ligament avulsion fracture from the tibial eminence Arthroscopy 2004 20 1 96 100 10.1016/j.arthro.2003.11.009 14716286

[B18] Kogan MG Marks P Amendola A Technique for arthroscopic suture fixation of displaced tibial intercondylar eminence fractures Arthroscopy 1997 13 3 301 6 10.1016/S0749-8063(97)90025-6 9195025

[B19] Schlummer T Klingelhöfer J Fortmeier B Giebel G Arthroscopically assisted refixation for avulsion fracture of the intercondylar eminence with Fiber-Wire cerclage Unfallchirurg 2004 107 6 525 31 15060774 10.1007/s00113-004-0752-8

